# Supplementary material for: Notch2-mediated plasticity between marginal zone and follicular B cells
Source: Nat Commun. 2021 Feb 17;12:1111. doi: 10.1038/s41467-021-21359-1 (PMC7889629; doi:10.1038/s41467-021-21359-1)
Supplement: Supplementary file 1 — Supplementary Information [file 41467_2021_21359_MOESM1_ESM.pdf]

Supplementary information

Notch2-mediated plasticity between Marginal Zone and Follicular B cells

Markus Lechner, Thomas Engleitner, Tea Babushku, Marc Schmidt-Supprian, Roland Rad, Lothar J. Strobl*, Ursula Zimber-Strobl*

*These authors equally supervised the work

Corresponding author: Ursula Zimber-Strobl

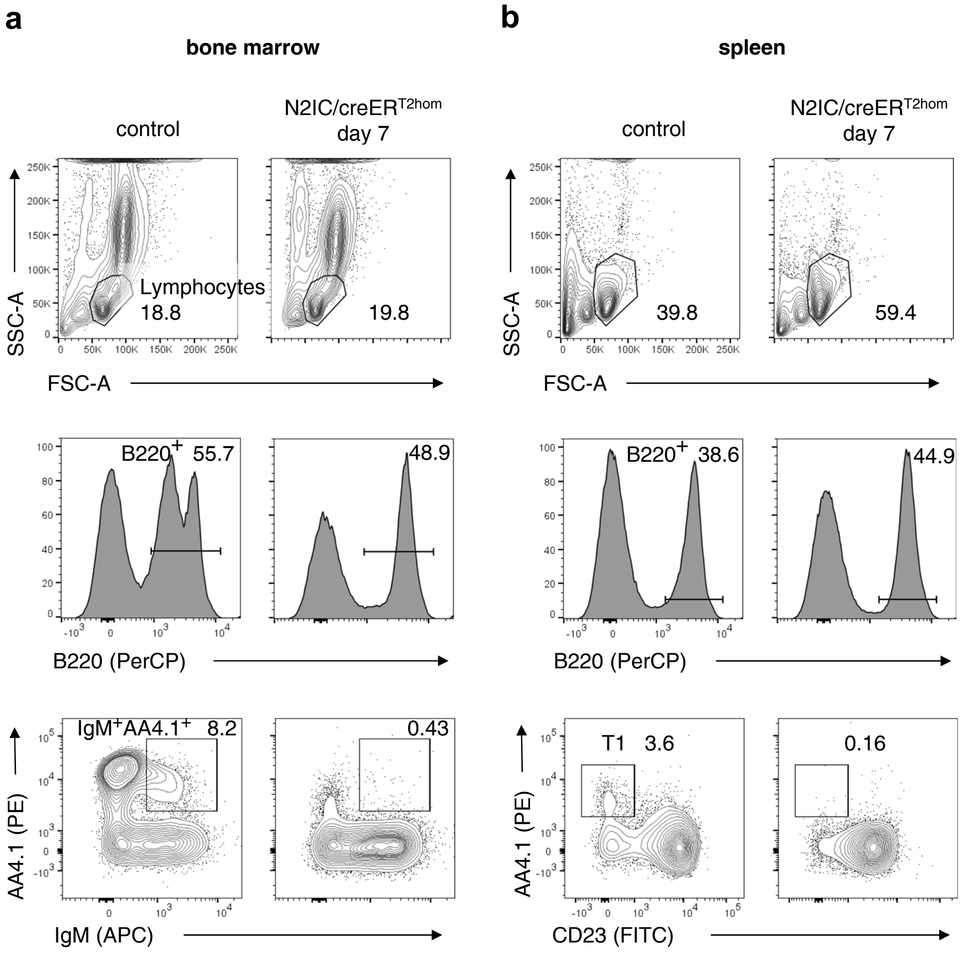

Supplementary Figure 1**. Pretreatment with 4x anti-IL7R antibody blocks B cell development in the bone marrow and transitional B cell influx into the spleen.** N2IC/creER^{T2hom} mice were treated as shown in Figure 1b. Flow cytometric analyses of bone marrow cells and splenocytes were performed 7 days after tamoxifen induction. **a** Analysis of the B cell development in the bone marrow: The latest development stage before efflux to the periphery, namely IgM^{+}AA4.1^{+}B220^{med} immature B cells, are absent after treatment with the anti-IL7R antibody. **b** Spleen: Subsequently, transitional (T1) B cells (B220^{+}AA4.1^{+}CD23^{-}) are absent in the spleen. FACS plots in A+B are gated on lymphocytes and B220^{+} cells. Data are representative for n=3 animals.

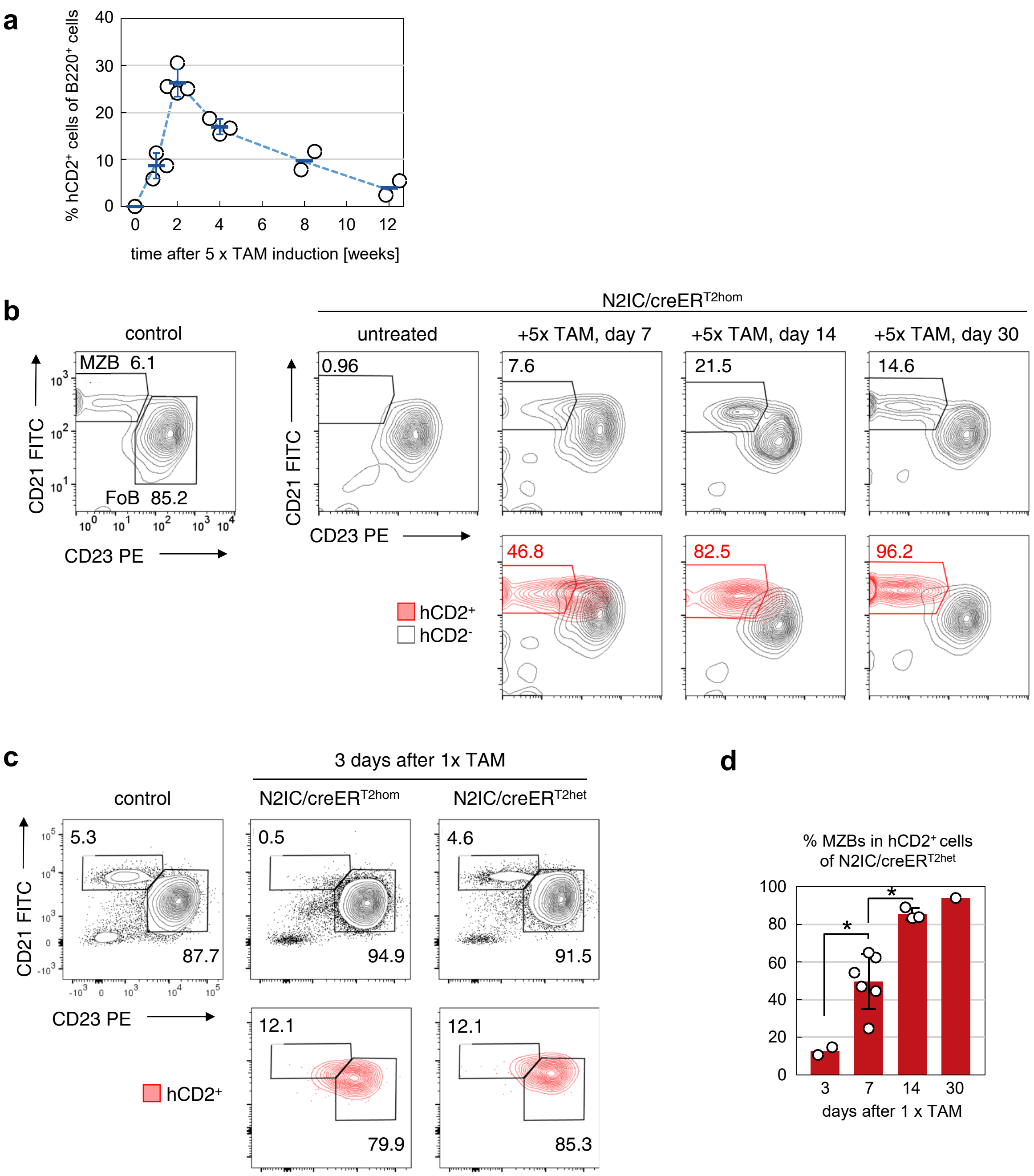

Supplementary Figure 2**. The FoB to MZB transition is not altered through repeated doses of tamoxifen or CD19 expression,** related to Figure 1**. a** Notch2IC/CreER^{T2hom} mice were treated with tamoxifen on 5 consecutive days (day 1-5). The graph shows data points from individual mice, mean percentages and SD values of hCD2^{+} B cells at the indicated time points after tamoxifen (TAM) treatment. n=3 (week 1), n=4 (week 2), n=3 (week 4) and n=2 (week 8 and week 12) mice **b** Representative FACS plots for the data points presented in **a**. Depicted is the CD21/CD23 phenotype of splenocytes from Notch2IC/CreER^{T2hom} mice at the indicated time points after 5x tamoxifen administration. The upper row is gated on lymphocytes and B220^{+} B cells. The bottom row is additionally separated into hCD2^{neg} (gray) and hCD2^{+} (red) B cells, indicated percentages refer to the percentage of MZB cells within total lymphocytes (black) and the fraction of hCD2^{+} cells (red). **c+d** Notch2IC-expressing B cells show a simlilar phenotype in CD19-deficient and –proficient cells: **c** Phenotype of hCD2^{+} B cells 3 days after 1x tamoxifen treatment in the indicated genotypes: The percentages of CD21^{high}CD23^{low} MZB cells were determined in CD21/CD23 FACS plots that were gated on B220^{+} (upper part) or B220^{+}hCD2^{+} cells (lower part) n=2. **d** The graph compiles the percentages of MZB cells within the fraction of hCD2^{+} B cells in N2IC/creER^{T2het} mice at the indicated time points after treatment with a single dose of tamoxifen. Bars indicate mean values and standard deviations at each time point. Data points represent values from individual mice for n=2 (day 3), n=6 (day 7), n=3 (day 14) and n=1 (day 30) mice. (*p=0.020 (d3 vs d7), *p=0.011 (d7 vs d14), ordinary one-way ANOVA, Tukey's multiple comparisons test).

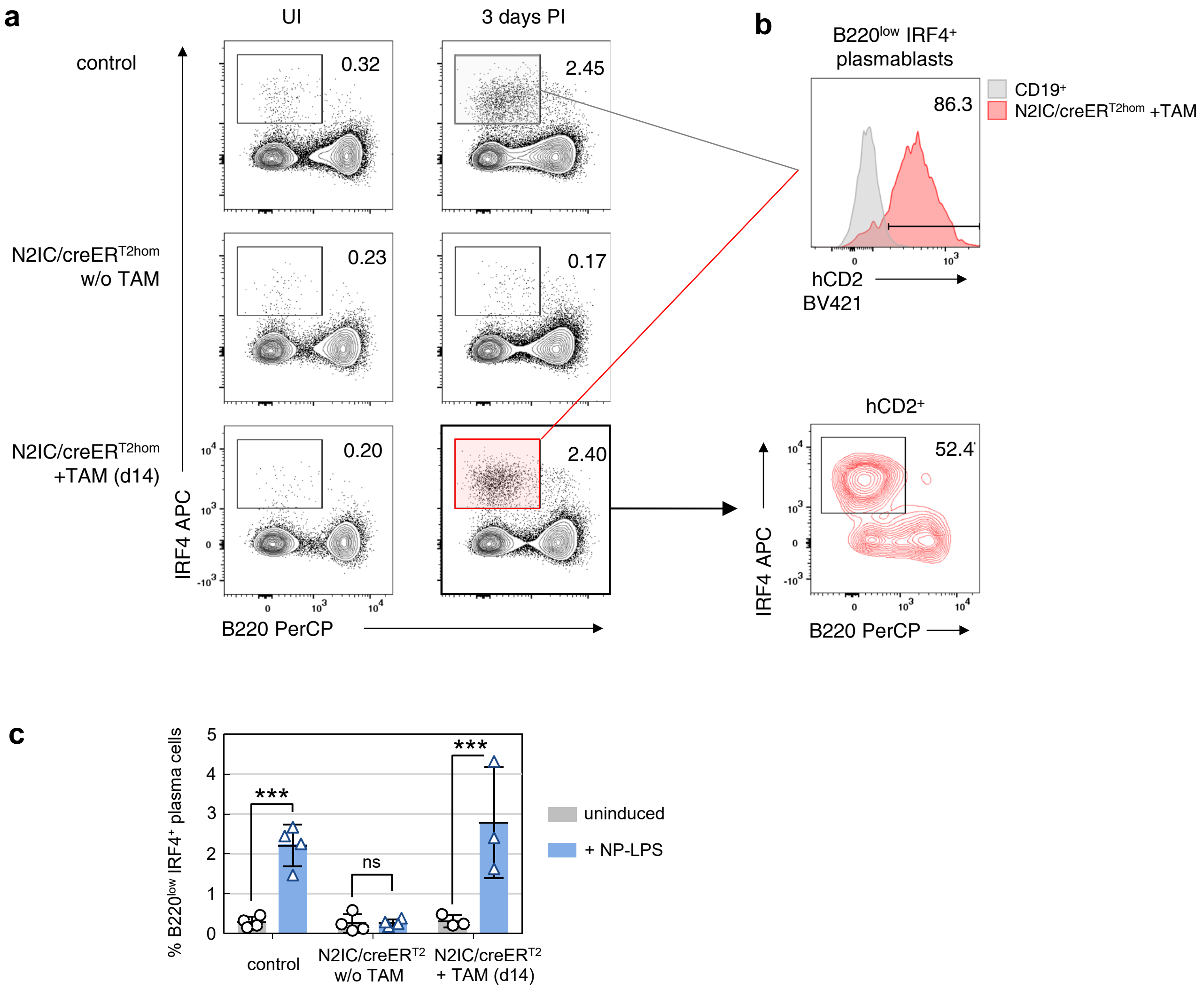

Supplementary Figure 3. **Reconstituted Notch2IC-expressing MZB cells respond to immunization with NP-LPS,** related to Figure 3**.**

a+b If not additionally mentioned, all FACS plots were pregated for living lymphocytes by FSC/SSC. **a** The percentages of plasmablasts (B220^{low}IRF4^{+}) were determined in the indicated genotypes three days after immunization with NP-LPS (3 days PI). As controls unimmunized mice (UI) are shown. **b** The regenerated plasmablast differentiation is exclusive to hCD2^{+} cells: The histogram (upper panel) shows an overlay of hCD2-expression in B220^{low}IRF4^{+} plasmablasts from immunized control (gray) and N2IC/creER^{T2hom} (+TAM (d14) mice (red). The number indicates the percentage of hCD2^{+} cells within plasmablasts of N2IC/creER^{T2hom} (+TAM) mice p.i.. The FACS plot (lower panel) illustrates the percentage of plasmablasts (IRF4^{high}B220^{low}) within the fraction of hCD2^{+} cells in immunized N2IC/creER^{T2hom} (+TAM) mice. **c** The graph compiles the percentages (mean and SD values) of B220^{low}IRF4^{+} plasmablasts within splenic lymphocytes in the indicated genotypes with (blue) and without (gray) immunization. **a-c** n=4 control mice and n=3 N2IC/creERT2 mice per treatment group were analyzed after two independent immunizations (***p=5.0E-04 (ctrl), ***p=1.7E-04 (N2IC+TAM), two-way ANOVA, Sidak's multiple comparisons test).

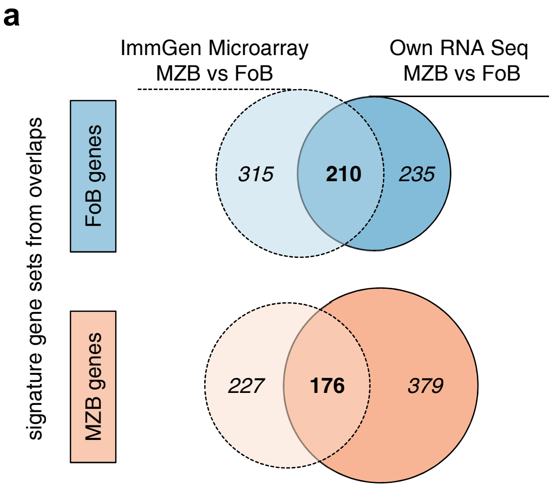

Supplementary Figure 4. **The enrichment of MZB signature genes increases in the time course of Notch2IC-induction,** related to Figure 4. **a** Venn diagrams illustrating the generation of MZB and FoB signature gene sets. **b** Pre-ranked Gene Set Enrichment Analysis (GSEA) using MZB genes (left column) or FoB genes (right column) sets and ranked gene expression data of all time points after tamoxifen treatment (day 3, 5, 7, 14) vs gene expression in hCD2^{-} cells. Enrichment scores (ES) are indicated for every plot. Nominal p values (NOM p-val.) and false discovery rates (FDR q-val) were <0,001 for all analyses.

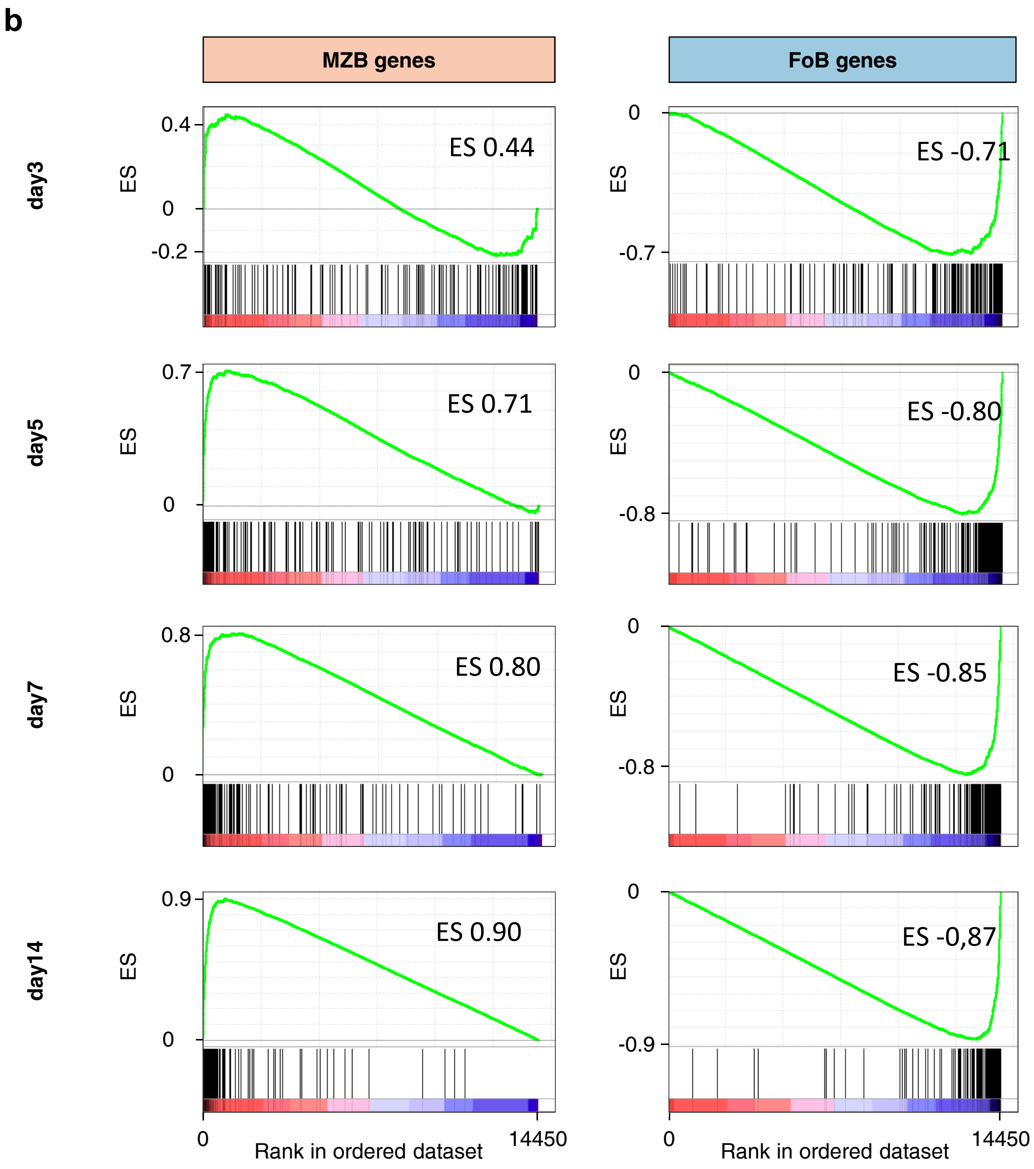

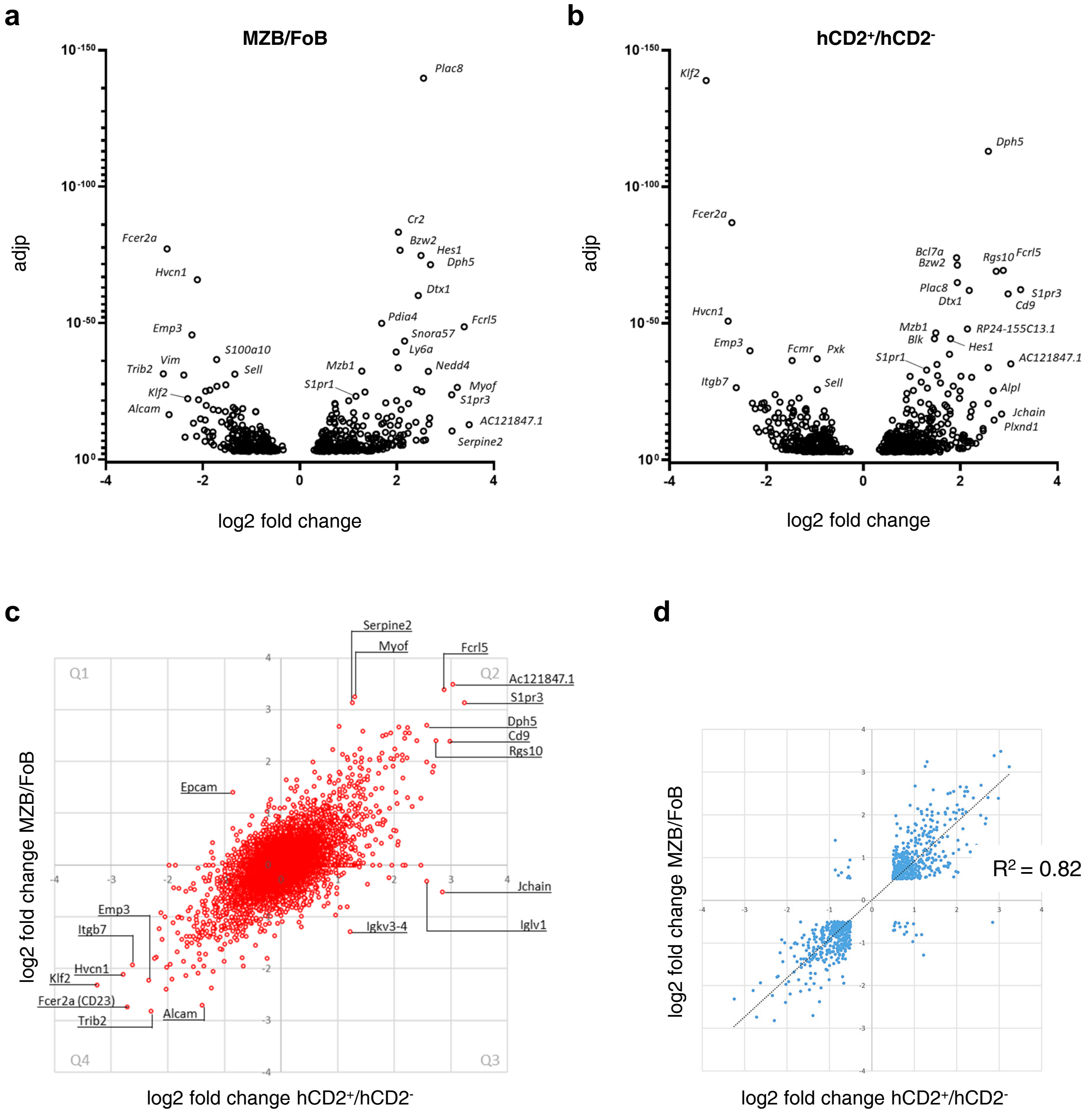

Supplementary Figure 5. **Comparison of differentially expressed genes between hCD2$^+$/hCD2$^-$ and FoB/MZB cells,** related to Figure 4**. a+b** Volcano plots of differentially expressed genes between wild type MZB versus FoB samples and hCD2$^+$ versus hCD2$^-$ cells from N2IC/creER^{T2hom} mice, 14 days after tamoxifen treatment. Plotted are log2-fold changes after shrinkage against adjusted p values (adjp). A cutoff was set at padj<0.001. 521 genes were differentially expressed with this high significance between MZB and FoB wild type samples **a**, and 732 genes between hCD2$^+$ and hCD2$^-$ cells **b**. **c+d** Correlation of differentially expressed genes of Notch2IC-expressing cells 14 days after TAM induction and wild type MZB versus FoB cells. Shrinked log2-fold gene expression values of all differentially expressed genes of sorted hCD2$^+$ (14 days after TAM) versus hCD2$^-$ cells (x-axis) and wild type MZB versus FoB cells (y-axis). **c** All n=14450 genes are plotted. Genes that were not detected in MZB vs FoB analysis (n=12113) were given the value 0. Top hits in both sample sets are additionally marked with gene names (in Q2 and Q4). Top inversely regulated genes are marked in Q1 and Q3. **d** Additional cut offs at +/- 0,5 log2-fold change were applied to data from (A). The resulting 915 shared genes strongly correlated ($p<0.0001$) in differential expression values, the R^2 value of the fitted linear regression trendline was added.

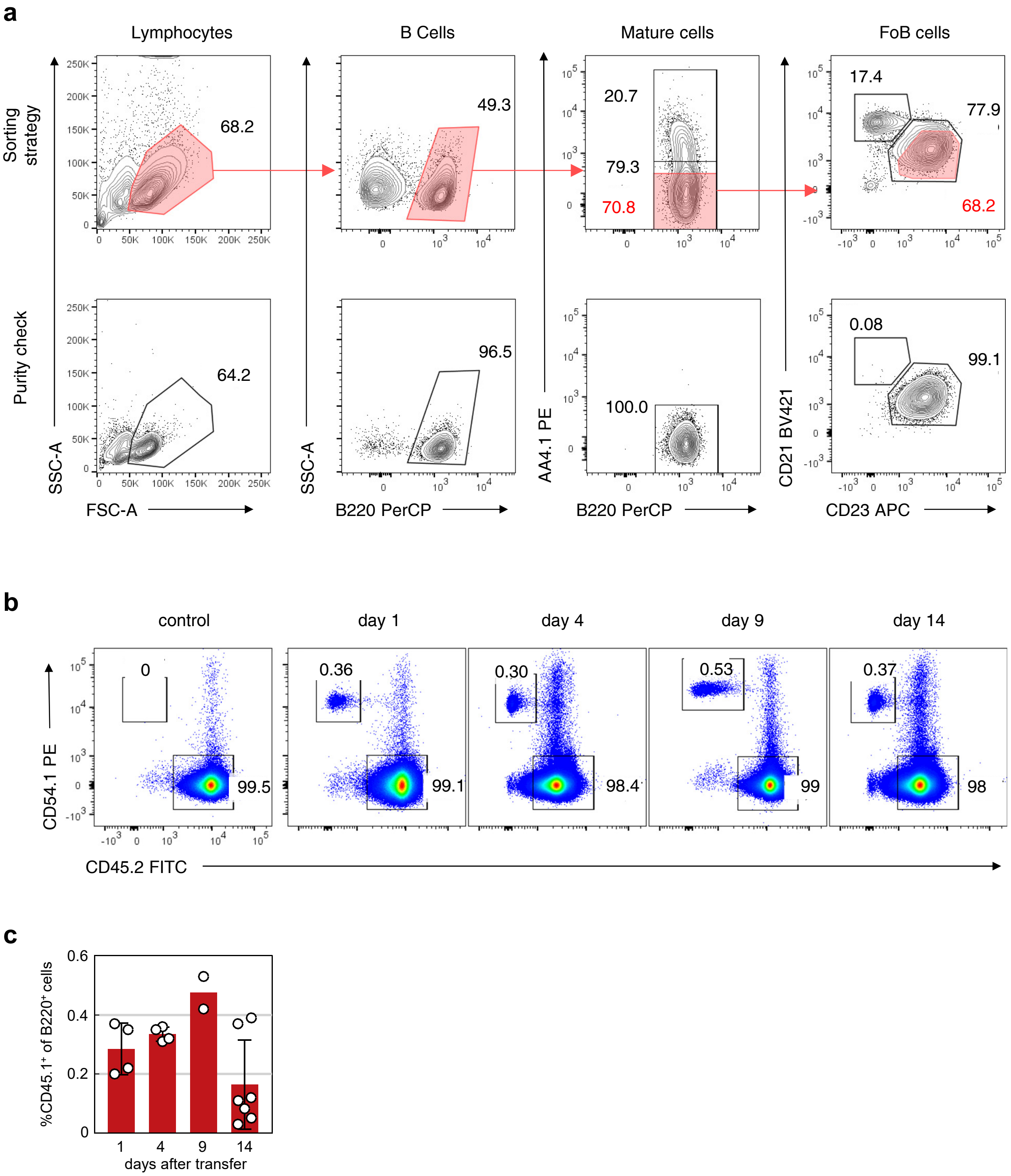

Supplementary Figure 6. **Sorting strategy of FoB cells from CD45.1 mice and additional analysis of the transplanted cells after adoptive transfer,** related to Figures 6 and 7**. a** Cell sorting and analysis of the purity: The hierarchical gating strategy is highlighted with red tinted gates. Analytical gates (black lines) were applied to verify the purity of the sorted FoB cells before transfer into congenic recipients. **b** Representative flow cytometric analysis of percentages of recovered CD45.1⁺ cells within splenic B220⁺ B cells in recipient mice at indicated time points after transplantation. **c** The graph compiles the percentages (mean and SD) of recovered CD45.1⁺ cells in the spleen at the indicated time points after transplantation. CD45.1 cells were gated as indicated in **b+c** n=4 (d1 and d4), n=2 (d9), n=7 (d14).

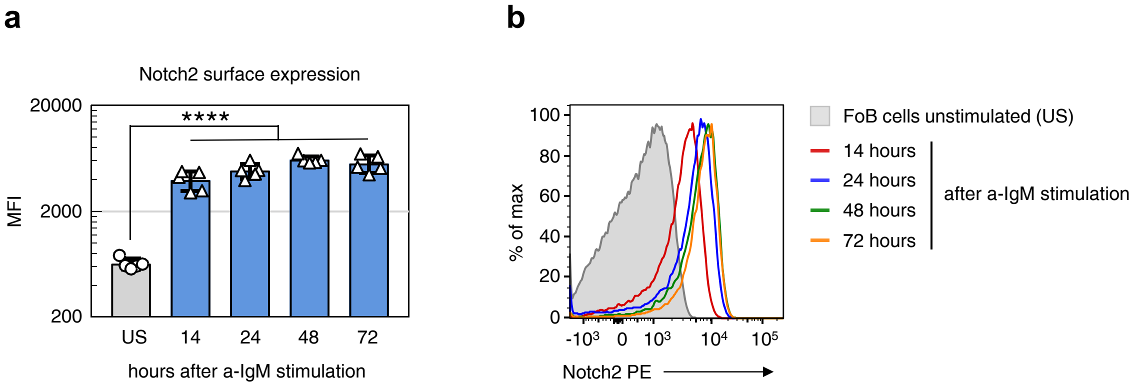

Supplementary Figure 7. **Notch2 is upregulated through BCR stimulation in vitro.**
Ex vivo isolated splenic FoB cells were cultured and stimulated with α-IgM. **a** The surface expression of Notch2 was determined by FACS after the indicated time points (US = unstimulated). The median fluorescent intensities (MFI) (mean and SD) are presented in the graph. (n=5 animals). (**** p=8.7E-13 (UI vs 14h), p=1.2E-13 (UI vs 24h), p=3.5E-14 (UI vs 48h), p=4.8E-14 (UI vs 72h), ordinary one-way ANOVA of logarithmized MFI values, Tukey's multiple comparisons test). **b** Representative FACS plot for the Notch2-expression at the cell surface, n=5.

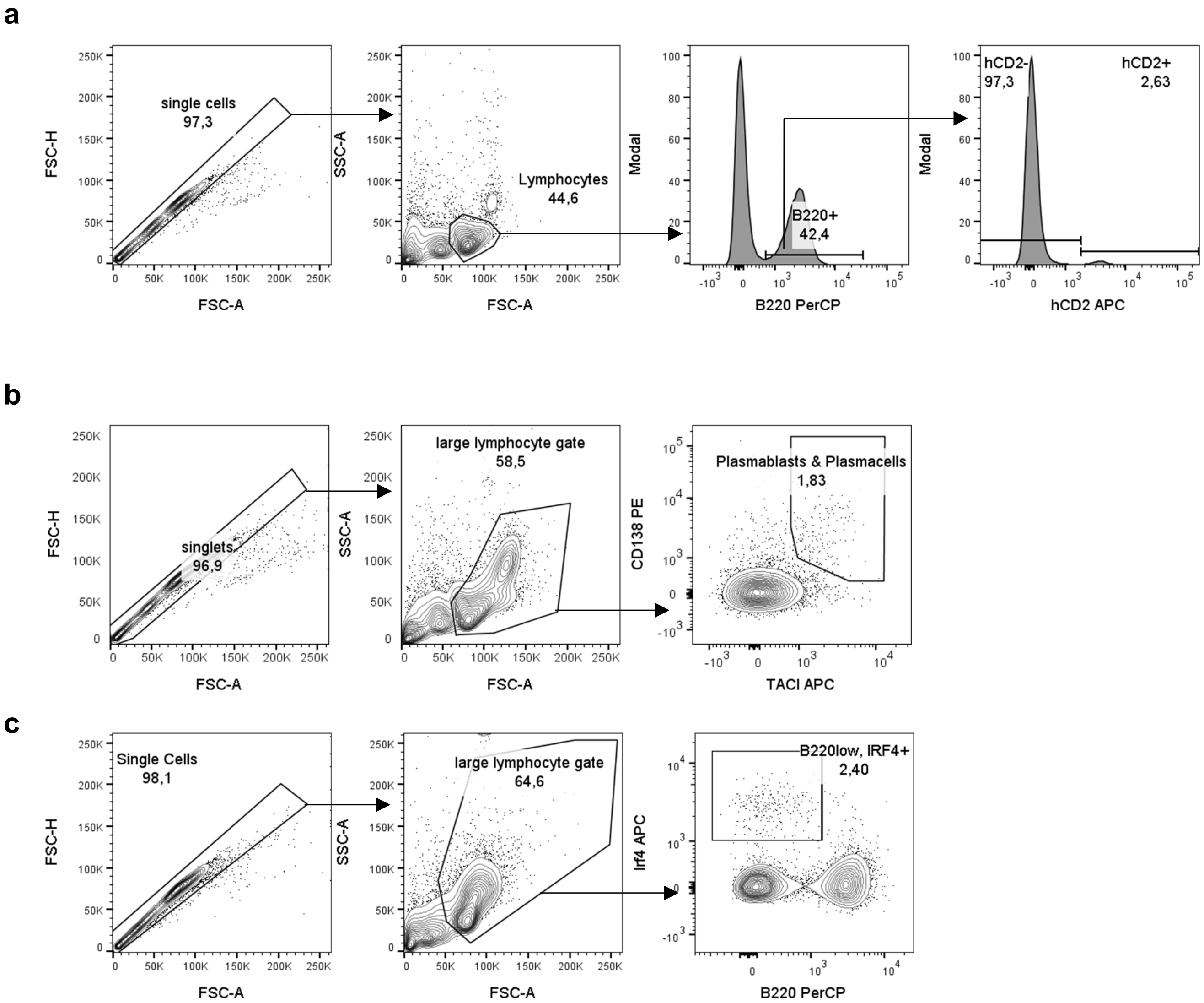

Supplementary Figure 8. **Additional representative flow cytometry gating strategies.**
a sequential gating strategy for B220⁺ B cells and hCD2⁺ B cells as used for Figure 1 and Figure S2. **b** sequencial gating strategy for plasmablasts used in Figure 3c-e and **c** intracellular staining for plasmablasts as used for Figure 4f and S3.

Supplementary Table 1

a

Serpine2	Cr2	Tmem131
Tmod2	Scd2	Fam129c
Ppl	Cdca7l	Abcg1
Zc3h12c	Kcnk5	Mxd4
Ffar2	Asap2	Pacsin2
Ccbp2	Dkkl1	Utrn
Srgap3	Ccr1	Ctsz
Gml	Cdc42bpb	Eef1b2
Clic4	Il9r	Taf1d
Tspan15	Pde8a	Edem1
Zfp532	Plxnc1	Adck3
Rap1gap	Tm6sf1	Rps6ka1
Trpm2	Hck	Ctsb
Ms4a7	Edaradd	Pdcd4
Alpl	Akr7a5	Pdlim2
Gpr156	Pdia4	Slc28a2
Myof	Myc	Mdfic
S1pr3	Pqlc2	Fgd2
Sema7a	Cdk5r1	Rplp1
Asb2	Grhpr	Phpt1
Fcrl5	Mfsd10	Rpl3
Adam28	Ell3	Rpl13
Cxcr7	Cacna1e	Pabpc4
Nebl	Slc29a3	Rpsa
Cd1d1	Impact	
Cdh17	Cebpb	
Plxnd1	Ptpn14	
Csf2rb	Alpk2	
Sorbs2	Ube2r2	
Smyd2	Ppfibp2	
Prdm1	Lpcat2	
Tsga13	Rrbp1	
Nrp2	Cd274	
Cd9	Abcg3	
Pde4d	Psap	
Dnase1l3	Nucb2	
Cyp39a1	Ptprj	
Pla2g7	Fuca2	
Adrbk2	Abhd6	
Ceacam2	Slc39a6	
Nedd4	Tmem154	
Ldlrad3	Rilpl2	
Atxn1	St3gal6	
Abcb1a	Tyrobp	
Tbc1d9	Entpd1	
Cd36	Lynx1	
Pik3r4	Rogdi	
Dph5	Gmnn	
Lair1	Cd86	
Ptpn22	Nab1	
Rgs10	Plbd1	
Pde4a	S1pr1	
Ccdc79	Traf1	
Abcb1b	Cbfa2t3	
Stom	Slc25a4	
Pmepa1	Rhbdf1	
Plac8	Cybb	
Mgst1	Gns	
Rsu1	Gadd45b	
Dtx1	Cyfip1	
Ahr	Plaur	
Dusp16	Gm2a	
Mpeg1	Sema4b	
Hes5	Trps1	
Marcks	Zfp239	
Tlr3	Crebl2	
Endod1	Cept1	
Inpp4a	Itm2c	
Hmgn3	Lax1	
Bst1	Cd81	
Bid	Sfxn1	
Tpst2	Lgmn	
Lysmd2	Wdfy2	
Grn	Arl5c	
Bzw2	Pip4k2a	
Ly6a	Blk	

b

Elmo1	Dock11	Tagln2
Smchd1	Tpm4	S1pr4
Calm2	Aff3	Arhgef3
Top2b	Gimap3	Stat4
Bptf	Sike1	Nsmce1
Atp2b1	Lfng	Sbk1
Tax1bp1	Tec	Cpm
Ywhaz	Fancm	Otud1
Ccdc50	Rasgrp3	Sfxn3
Irf2	Lbh	Insr
Snx2	Icosl	Cnn3
Eml4	Fgd3	Bbs9
Slc4a7	Gimap4	Atp1b1
Stk17b	Map4k4	Arhgef18
Rapgef6	Tnik	Gpr146
Stk24	Ssh2	Slc15a3
Ankrd11	Satb1	Rnf144a
Crlf3	Sorl1	Fry
Gpatch8	Plec	Ms4a4c
Aff4	Ifnar1	S100a10
Ap1m1	B3gnt8	Cdkl1
Dgkd	Klf3	Vpreb3
Man1a	Cd83	B3gnt5
Jak1	Dgka	Ssbp2
Dek	H3f3a	Baiap2
Grk6	Spns2	Xylt1
Ankrd44	Neurl3	Gmfg
Rfc1	Tcp11l2	Zfp318
Cdk19	St6gal1	Sfn
Mylip	Fam107b	Bcl6
Bend5	Rbm38	Tctn1
Msn	Dcaf12	Enpp1
Fmnl1	Kif21b	Csrp2
Icam2	Rara	Itgb7
Ints4	Rhoh	Gadd45g
Mtss1	Add3	Hvcn1
Rad17	Atp6v0a1	Ahnak
Gpr183	Cytip	Sgk1
Dnajc9	Dusp3	Bach2
Cdc42se1	Sh3bp5	Slamf1
Myh9	Rasa3	Cd55
Aim1	Fli1	Emp3
Rap1b	Rassf3	Fam101b
Wasf2	Fam65b	Nab2
Mier1	Btla	Fam46a
Lsp1	Sepp1	Mapk12
Dmxl1	Tsc22d3	Vim
Smap2	Cdkn2d	Klf2
Nuak2	Ehd4	Car2
Pde4b	Rabgef1	Fcgrt
Card6	Ccr7	Kctd14
Med13	Il21r	Fcer2a
Ciita	Samhd1	Rgl1
Mef2c	Cd69	Mapk11
Stap1	Cerk	Cacna1i
St3gal1	Abca1	Trib2
Lmbrd1	Sell	Serinc5
Chst3	Cxcr4	Alcam
Parp1	Cmah	Dusp4
Prkd2	Flna	
Iqgap1	Il6ra	
Skil	Pgap1	
Ptma	Fam69a	
Ccr6	Slc14a1	
Ccm2	Fchsd2	
Serpinb1a	Lasp1	
Plcl2	Fxyd5	
Chst15	Zfp608	
Hdac9	Trim59	
Hexb	Add1	
Snx29	Tuba1a	
Tbc1d1	Cd200	
Ppp3ca	Lmo2	
Akt3	Rnf122	
Csnk1g3	Maml3	
Pxk	Il4ra	

Supplementary Table 1: MZB and FoB signature gene sets used for GSEA analyses.
a “MZB genes“. **b** “FoB genes“

Supplementary Table 2

a	b	
1500011K16	1110038D17Rik	Peli1
Rik	Fam46a	Spns2
Myl4	Lrrc8c	Cdc42se1
2010012O05	Rdh12	Ikbkb
Rik	Adamts6	Pgap1
Myof	Fam65b	Ssbp2
3830406C13	Lsp1	Cdkn2d
Rik	Rfk	IL27Ra
Nedd4	Add3	Pglyrp1
A930005H10	Fam69a	St3gal1
Rik	Madd	ceacam1
Oosp1	Rftn2	Ints4
Akr1e1	Adrb2	Phyhd1
Pdzd2	Fcgrt	Stac2
As3mt	Mapk11	ceacam2
Per3	Rnf144a	ITGA6
Asb2	AHNAK	Pira2
Plekhg3	Flna	STAT4
BC016495	Marcks11	Ckap4
Pmepa1	S1PR4	ITGB7
Cacna1e	AI467606	Pkn2
Ptpn14	Fry	Stk38
Ccr5	Marveld2	Dgka
PtpN22	Sbk1	Jhdm1d
CD36	Aida	Plekhf2
Ptprj	Fuca1	Tagln2
Cdk5r1	Mex3b	Dtx1
Rab37	SELL	Kctd14
Cyp39a1	Arhgap5	Plk2
Rcn1	GADD45g	Tcp11l2
D8Ertd82e	Mllt6	DUSP10
Rgs18	Sfxn3	KLF3
Derl3	Arhgef18	Pml
S1pr3	Gimap4	Tiparp
Dtx3	Msi2	DUSP3
Sema4b	Sgk1	KLF3
Edaradd	Atp1b1	Pold1
Sema7a	Gm527	Tle1
Ehd4	Mtap	Emp3
Spns3	Sh3bp5	Lasp1
Fam92a	Atp2b1	Prkcb
Tbc1d19	Gna15	Tmod3
Fbxw17	Myadm	Enpp1
Tmem18	Slc2a6	Lfng
Fuca2	Ccdc52	Rab6b
Tmem26	Gpr146	Trib2
Gadd45b	Nap1l1	ENSMUSG0000007
Tnfrsf21	Slc40a1	4420
Gramd1b	Ccdc88c	Lgals1
Top2a	Gramd4	Rabgef1
Hbb-b1	Nap1l1	Ttc13
Trem1	Slc43a1	Evi5
Hck	CD200	Lilrb3
Trim32	Gvin1	Rasa3
Hpse	Nrm	Txndc5
Trps1	Slfn5	Fam101b
Lpcat2	CD46	Lmo2
Ttc28	Hes1	Rasgrp2
Ltk	P2ry10	Vim
Tubg2	Smad1	Fam167a
Lynx1	CD55	Lrrc8a
Tyrobp	Hexb	Rassf3
Marcks	Pde2a	Zfp53ar
Ube2e3	Spn	
Mdfic	cdc2l6	
Vwa5a	Hsd11b1	

Supplementary table 2: KLF2-knockout gene sets used for GSEA analysis in Figure 5b. **a** “KLF2-KO up” and **b** “KLF2-KO down” gene sets. Differentially expressed genes between CD19Cre/Klf2$^{fl/fl}$ and CD19Cre/Klf2$^{+/+}$ FoB cells were extracted from Hart et al. (2011), Supplementary Figure 8
