# Supplementary material for: Notch2-mediated plasticity between marginal zone and follicular B cells
Source: Nat Commun. 2021 Feb 17;12:1111. doi: 10.1038/s41467-021-21359-1 (PMC7889629; doi:10.1038/s41467-021-21359-1)
Supplement: Supplementary file 3 — Reporting Summary [file 41467_2021_21359_MOESM3_ESM.pdf]

nature research

Corresponding author(s): Ursula Zimber-Strobl

Last updated by author(s): Jan 5, 2021

Reporting Summary

Nature Research wishes to improve the reproducibility of the work that we publish. This form provides structure for consistency and transparency in reporting. For further information on Nature Research policies, see our Editorial Policies and the Editorial Policy Checklist.

Statistics

For all statistical analyses, confirm that the following items are present in the figure legend, table legend, main text, or Methods section.

n/a	Confirmed	
☐	☒	The exact sample size (*n*) for each experimental group/condition, given as a discrete number and unit of measurement
☐	☒	A statement on whether measurements were taken from distinct samples or whether the same sample was measured repeatedly
☐	☒	The statistical test(s) used AND whether they are one- or two-sided<br>*Only common tests should be described solely by name; describe more complex techniques in the Methods section.*
☒	☐	A description of all covariates tested
☒	☐	A description of any assumptions or corrections, such as tests of normality and adjustment for multiple comparisons
☐	☒	A full description of the statistical parameters including central tendency (e.g. means) or other basic estimates (e.g. regression coefficient) AND variation (e.g. standard deviation) or associated estimates of uncertainty (e.g. confidence intervals)
☐	☒	For null hypothesis testing, the test statistic (e.g. *F*, *t*, *r*) with confidence intervals, effect sizes, degrees of freedom and *P* value noted<br>*Give P values as exact values whenever suitable.*
☒	☐	For Bayesian analysis, information on the choice of priors and Markov chain Monte Carlo settings
☒	☐	For hierarchical and complex designs, identification of the appropriate level for tests and full reporting of outcomes
☒	☐	Estimates of effect sizes (e.g. Cohen's *d*, Pearson's *r*), indicating how they were calculated

Our web collection on statistics for biologists contains articles on many of the points above.

Software and code

Policy information about availability of computer code

Data collection	Dropseq tools v1.13,<br>DESeq2 v1.8,<br>Reference genome GRCm38, Gencode gene annotation release M19,<br>GSEA v4.0.2 and v4.0.1<br>Leica Application Suite X<br>ImmGen (http://rstats.immgen.org/PopulationComparison/index.html)
Data analysis	R v3.4.4<br>GraphPad Prism8<br>MS Excel 2016<br>ImageJ 1.46r<br>FlowJo V10

For manuscripts utilizing custom algorithms or software that are central to the research but not yet described in published literature, software must be made available to editors and reviewers. We strongly encourage code deposition in a community repository (e.g. GitHub). See the Nature Research guidelines for submitting code & software for further information.

Data

Policy information about availability of data

All manuscripts must include a data availability statement. This statement should provide the following information, where applicable:

- Accession codes, unique identifiers, or web links for publicly available datasets
- A list of figures that have associated raw data
- A description of any restrictions on data availability

The RNA seq datasets generated and analyzed in this study are available through ENA accession code "PRJEB35207 (https://www.ebi.ac.uk/ena/browser/view/PRJEB35207)". Any additional data presented in this paper is available from the corresponding author upon request. Source data is provided with this paper as Excel file and includes Figures 1c, 1e, 1g, 1h, 3d, 3f, 6b, 6c, 7a and Supplementary Figures 2a, 2d, 3c, 6c and 7. For generation of the MZB signature gene sets published microarray data (GSE11961) were processed by the population comparison tool from www.immgen.org.

Field-specific reporting

Please select the one below that is the best fit for your research. If you are not sure, read the appropriate sections before making your selection.

- [x] Life sciences
- [ ] Behavioural & social sciences
- [ ] Ecological, evolutionary & environmental sciences

For a reference copy of the document with all sections, see nature.com/documents/nr-reporting-summary-flat.pdf

Life sciences study design

All studies must disclose on these points even when the disclosure is negative.

Sample size	Sample sizes for the in vivo studies were designed to obtain statistically significant results while maintaining animal numbers as low as possible. Sample size numbers were also based on previous experiments or on preliminary data from our laboratory.
Data exclusions	no data were excluded
Replication	All in vivo experiments were performed with at least 3 biological replicates per time point, treatment or transplantation. In vitro experiments were performed in duplicates. Replications of in vivo and in vitro experiments were successful.
Randomization	Randomization for RNA Seq Experiments: All samples were frozen and stored after sort. RNA-purification of samples was performed in randomized groups of 6-8 samples at once. All samples were handed to cooperation partners as randomized numbered list for library preparation and RNA Seq. For all in vivo and in vitro studies, mice were allocated in cohorts based on genotype or time points after tamoxifen treatment and no randomization was performed. Mice used in the study were maintained at minimally perturbative conditions. Control and analytical mice were analysed in parallel. The experiments were repeated with different animals and independent treatments with tamoxifen, independent immunizations and independent adoptive transfer experiments.
Blinding	Investigators performing the RNA Sequencing experiment and analysis were blinded. In all other experiments, the investigator was not blinded as phenotypic observations in FACS and microscopy were clearly visible and consistent. Data analysis of FACS were performed unbiased with consistent gating for all genotypes and time points.

Reporting for specific materials, systems and methods

We require information from authors about some types of materials, experimental systems and methods used in many studies. Here, indicate whether each material, system or method listed is relevant to your study. If you are not sure if a list item applies to your research, read the appropriate section before selecting a response.

Materials & experimental systems

n/a	Involved in the study	
[ ]	[x]	Antibodies
[x]	[ ]	Eukaryotic cell lines
[x]	[ ]	Palaeontology and archaeology
[ ]	[x]	Animals and other organisms
[x]	[ ]	Human research participants
[x]	[ ]	Clinical data
[x]	[ ]	Dual use research of concern

Methods

n/a	Involved in the study	
[x]	[ ]	ChIP-seq
[ ]	[x]	Flow cytometry
[x]	[ ]	MRI-based neuroimaging

Antibodies

Antibodies used	Anti-Human CD2 APC (clone RPA-2.10) BD Biosciences Cat#560642; RRID:AB_1727443<br>Anti-Human CD2 BV421 (clone RPA-2.10) BD Biosciences Cat#562667; RRID:AB_2737708

Anti-Human CD2 FITC (clone RPA-2.10) BD Biosciences Cat#561759; RRID:AB_10896133
Anti-Mouse CD45R/B220 PerCP (clone RA3-6B2) BD Biosciences Cat#553093; RRID:AB_394622
Anti-Mouse CD21/CD35 FITC (clone 7G6) BD Biosciences Cat#561769; RRID:AB_10924591
Anti-Mouse CD21/CD35 BV421 (clone 7G6) BD Biosciences Cat#562756; RRID:AB_2737772
Anti-Mouse CD23 PE (clone B3B4) BD Biosciences Cat#561773; RRID:AB_10895122
Anti-Mouse CD23 APC (clone EBVCS2) eBioscience Cat#17-0238; RRID:AB_469364
Anti-Mouse CD97 PE (clone AA4.1) eBioscience Cat#12-5892; RRID:AB_466018
Anti-Mouse CD1d PE (clone 1B1) BD Biosciences Cat#553846; RRID:AB_2073521
Anti-Mouse CD1d AF647 (clone 1B1) Biolegend Cat#123511; RRID:AB_1236539
Anti-Mouse IgM BV450 (clone R6-60.2) BD Biosciences Cat#560575; RRID:AB_1645286
Anti-Mouse IgM APC (clone II/41) BD Biosciences Cat#550676; RRID:AB_398464
Anti-Mouse IgD FITC (clone 11-26c.2a) BD Biosciences Cat#553439; RRID:AB_394859
Anti-Mouse CD138 PE (clone 281-2) BD Biosciences Cat#553714; RRID:AB_395000
Anti-Mouse IRF4 APC (clone 3E4) eBioscience Cat#50-9858; RRID:AB_2574393
Anti-Mouse IgG1 PE (clone A85-1) BD Biosciences Cat#550083; RRID:AB_393553
Anti-Mouse TACI APC (clone ebio8F10-3) eBioscience Cat#17-5942; RRID:AB_842758
Anti-Mouse CD19 PE Vio770 (clone REA749) Miltenyi Cat#130-112-037; RRID:AB_2655830
Anti-Mouse Notch2 PE (clone HMN2-35) Biolegend Cat#130707; RRID:AB_1227725
Anti-Mouse CD45.2 FITC (clone 104) Tonbo Cat#35-0454; RRID:AB_2621692
Anti-Mouse CD45.1 PE (clone A20) Tonbo Cat#50-0453; RRID:AB_2621765
For IHC:
Anti-Laminin (clone L9393) Sigma-Aldrich Cat#L9393; RRID:AB_477163
Anti-Human CD2 Biotin (clone RPA-2.10) BD Biosciences Cat#555325; RRID:AB_395732
Anti-Mouse CD90.2 (Thy1.2) Biotin (clone 30-H12) BD Biosciences Cat#553011; RRID:RRID:AB_394549
Anti-MOMA-1 FITC Abcam Cat#ab34355
Anti-Mouse IgD FITC (clone 11-26c.2a) BD Biosciences Cat#553439; RRID:AB_394859
Anti-Mouse IgM (clone 1020-01) Southern Biotech Cat#1020-01; RRID:AB_2794197
Anti-Goat IgG AF647 Invitrogen Cat#A-21469; RRID:AB_2535872
Anti-Rabbit IgG Peroxidase Sigma-Aldrich Cat#A0545; RRID:AB_1645286

Anti-Mouse IL7R (clone A7R34) was produced in house

Validation

All antibodies were validated by the manufacturers for antigen specificity and species reactivity as shown in the data sheets and attached references for each catalogue number. Antibodies were additionally validated for specificity and sensitivity and titrated in pre-experiments by using positive and negative control samples.

Animals and other organisms

Policy information about studies involving animals; ARRIVE guidelines recommended for reporting animal research

Laboratory animals

Mouse: Notch2ICSTOPfl (Hampel et al., 2011)
Mouse: R26/CAG-CARd1STOPfl (Heger et al., 2015)
Mouse: CD19creERT2 (Yasuda et al., 2013)
Mouse: B6.SJL-Ptprca Pepcb/BoyJ (CD45.1), JAX Stock No 002014
Mouse: C57BL/6J wild type CD45.2, JAX Stock No 000664
Male and female mice at the age of 10-20 weeks were used for most experiments.
Only female mice were used for RNA Seq.

Wild animals

No wild animals were used in this study.

Field-collected samples

No field-collected samples were used in this study.

Ethics oversight

Animals were bred and maintained in specific pathogen-free conditions and experiments were performed in compliance with the German Animal Welfare Law and were approved by the Institutional Committee on Animal Experimentation and the government of Upper Bavaria.

Note that full information on the approval of the study protocol must also be provided in the manuscript.

Flow Cytometry

Plots

Confirm that:

- [x] The axis labels state the marker and fluorochrome used (e.g. CD4-FITC).
- [x] The axis scales are clearly visible. Include numbers along axes only for bottom left plot of group (a 'group' is an analysis of identical markers).
- [x] All plots are contour plots with outliers or pseudocolor plots.
- [x] A numerical value for number of cells or percentage (with statistics) is provided.

Methodology

Sample preparation	Single cell suspensions were generated from spleen and bone marrow. Surface stainings of lymphocytes were performed on ice for 20 minutes in MACS buffer (Miltenyi). For intracellular FACS-stainings, cells were fixed in 2% formaldehyde (1:2 PBS diluted Histofix) for 5 minutes at room temperature and permeabilized in ice-cold 100% methanol for 10 minutes on ice. Cells were stained for 45 minutes at room temperature with the corresponding antibodies
Instrument	FACS Calibur, LSR Fortessa, FACS Aria III
Software	FlowJo V10
Cell population abundance	post-sort analysis are plotted in the supplement figures. purified FoB cells were always >99%
Gating strategy	Gating of lymphocytes is shown in Figure S1A, S6A and S8. Important gating strategies for analysis of cells are listed in figures S1 and S8. Gating for cell sorting is indicated in Figure S6A

- [x] Tick this box to confirm that a figure exemplifying the gating strategy is provided in the Supplementary Information.
